# PINK1 Deficiency Attenuates Astrocyte Proliferation Through Mitochondrial Dysfunction, Reduced AKT and Increased p38 MAPK Activation, and Downregulation of EGFR

**DOI:** 10.1002/glia.22475

**Published:** 2013-02-26

**Authors:** Insup Choi, Jun Kim, Hey-Kyeong Jeong, Beomsue Kim, Ilo Jou, Sang Myun Park, Linan Chen, Un-Jung Kang, Xiaoxi Zhuang, Eun-hye Joe

**Affiliations:** 1 Neuroscience Graduate Program, Ajou University School of MedicineSuwon, Korea; 2 Department of Pharmacology, Ajou University School of MedicineSuwon, Korea; 3 Chronic Inflammatory Disease Research Center, Ajou University School of MedicineSuwon, Korea; 4 Department of Neurobiology, University of ChicagoChicago, IL; 5 Department of Neurology, University of ChicagoChicago, IL; 6 Brain Disease Research Center, Ajou University School of MedicineSuwon, Korea

**Keywords:** PINK1, astrocyte, proliferation, Parkinson's disease

## Abstract

PINK1 (PTEN induced putative kinase 1), a familial Parkinson's disease (PD)-related gene, is expressed in astrocytes, but little is known about its role in this cell type. Here, we found that astrocytes cultured from PINK1-knockout (KO) mice exhibit defective proliferative responses to epidermal growth factor (EGF) and fetal bovine serum. In PINK1-KO astrocytes, basal and EGF-induced p38 activation (phosphorylation) were increased whereas EGF receptor (EGFR) expression and AKT activation were decreased. p38 inhibition (SB203580) or knockdown with small interfering RNA (siRNA) rescued EGFR expression and AKT activation in PINK1-KO astrocytes. Proliferation defects in PINK1-KO astrocytes appeared to be linked to mitochondrial defects, manifesting as decreased mitochondrial mass and membrane potential, increased intracellular reactive oxygen species level, decreased glucose-uptake capacity, and decreased ATP production. Mitochondrial toxin (oligomycin) and a glucose-uptake inhibitor (phloretin) mimicked the PINK1-deficiency phenotype, decreasing astrocyte proliferation, EGFR expression and AKT activation, and increasing p38 activation. In addition, the proliferation defect in PINK1-KO astrocytes resulted in a delay in the wound healing process. Taken together, these results suggest that PINK1 deficiency causes astrocytes dysfunction, which may contribute to the development of PD due to delayed astrocytes-mediated repair of microenvironment in the brain.

## INTRODUCTION

Mutations in PINK1 (PTEN induced putative kinase 1), a familial Parkinson's disease (PD)-related gene, cause an autosomal recessive and early onset PD (Valente et al., 2001). PINK1 contains an N-terminal mitochondrial targeting sequence and a serine/threonine kinase domain that has homology to calcium/calmodulin-regulated kinase 1 (CAMK1). The importance of PINK1 in mitochondrial function has been inferred from its localization in and processing by mitochondria (Lin and Kang, 2008, 2010; Mills et al., 2008; Valente et al., 2004; Zhou et al., 2008). Accordingly, PINK1 knockdown (KD) or expression of kinase-dead PINK1 mutants in PC12 and SH-SY5Y cells decrease ATP generation and oxygen consumption (Beilina et al., 2005; Liu et al., 2009; Sim et al., 2006) and increases ROS production (Gandhi et al., 2009). In addition, PINK1-KD and -knockout (KO) cells, including neurons, are more vulnerable to various insults than wild-type (WT) cells (Deng et al., 2005; Haque et al., 2008). However, animal models that carry a PINK1 mutation do not develop PD-like symptoms, such as degeneration of dopaminergic neurons and Lewy body formation (Gispert etal., 2009; Kitada etal., 2007). Therefore, the emerging concept of the onset and progression of dopaminergic neuronal degeneration *in vivo* is that certain environmental factors must cooperate with genetic factors in the development of PD (Dawson et al., 2010).

Astrocytes, the most abundant cell types in the brain, express PINK1 (Gandhi et al., 2006; Wilhelmus etal., 2011). However, it is not known what roles PINK1 plays in astrocytes, and how astrocyte function is altered by PINK1 mutation. Astrocytes maintain homeostasis of the brain, by regulating extracellular levels of glutamate, ion concentration and pH (Anderson and Swanson, 2000; Simard and Nedergaard, 2004), and supplying energy in the form of lactate (Brown and Ransom, 2007; Chih and Roberts, 2003; Pellerin etal., 2007). In the injured brain, astrocytes proliferate and protect neurons by isolating injury sites, preventing oxidative stress (Wilson, 1997), and inhibiting excessive inflammation (Kim et al., 2010; Min et al., 2006; Yang et al., 2007). Further-more, astrocytes contribute to the repair of the injured brain by regulating extracellular matrix proteins and growth factors that support axonal growth (Costa et al., 2002; Tom et al., 2004; White and Jakeman, 2008). Therefore, the loss of astrocyte function could affect the development of neurodegeneration.

This study suggests that PINK1 deficiency causes a defect in the proliferative response of astrocytes to epidermal growth factor (EGF) and fetal bovine serum (FBS) and this defect leads to delayed wound-healing processes. The proliferation defect in PINK1-KO astrocytes appeared to be caused by mitochondrial dysfunction through an increase in p38 MAPK (mitogen-activated protein kinase) activation and a decrease in AKT activation and EGF receptor (EGFR) expression. Therefore, PINK1 deficiency may cause a delay in the repair of the damaged brain, which could contribute to the development of neurodegeneration.

## MATERIALS AND EXPERIMENTAL METHODS

### Materials

EGF was purchased from Peprotech (Rocky Hill, NJ). SB203580 was from Enzo Life Sciences International (Plymouth Meeting, PA). MitoTracker Red CMXRos, MitoTracker Green FM, and carboxyl-H2DFFDA were from Invitrogen (Carlsbad, CA). Oligomycin, phloretin, *N*-acetyl-l-cysteine (NAC), and other reagents were from Sigma (St. Louis, MO).

### Animals

PINK1-deficient mice were generated by replacing a 5.6-kb genomic region of the PINK1 locus, including exons 4–7 and the coding portion of exon 8, with a PGK-neo-polyA selection cassette flanked by FRT sequences, as previously described (Xiong et al., 2009). Both the 3.5 and 4.8-kb homologous arms were amplified by polymerase chain reaction (PCR), using genomic DNA isolated from E14Tg2A.4 embryonic stem cells (BayGenomics) as a template. E14Tg2A.4 em-bryonic stem cells were electroporated (800 V and 3 μF) with30 μg of linearized targeting construct. G418-resistant clones were screened by Southern blotting for homologous recombination with a 5′ external probe. Positive cells were injected into C57BL6/J blastocysts to generate chimeras, which were then mated with C57BL6/J WT mice to generate heterozygotes. Heterozygous mice on a 129 × C57BL/6 mixed background were bred to generate PINK1-null mice and their WT littermate controls for experiments. Mice were genotyped by multiplex PCR of genomic DNA extracted from tail snips. The first primer pair amplified part of intron 6 of PINK1 (present in all mice); the second primer pair amplified part of neor (absent in WT mice); and the third primer pair amplified intron 6 of PINK1 (absent in homozygous mutants). The absence of PINK1 expression was verified by reverse transcription-PCR (RT-PCR) and in situ hybridization (data not shown). All animal procedures were approved by the Institutional Animal Care and Usage Committee of theUniversity of Chicago and the Ajou University School of Medicine Ethics Review Committee for Animal Research (Amc-28).

### Cell Culture

**FIGURE 1:**
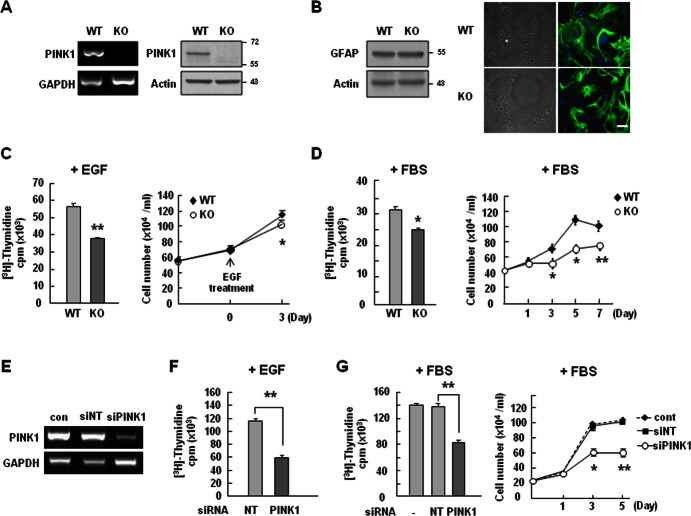
PINK1 deficiency causes a proliferation defect. (**A**) The absence of PINK1 expression in astrocytes of PINK1-KO mice was confirmed by RT-PCR (left panel) and Western blot (right panel). (**B**) GFAP expression and morphology were not different in astrocytes prepared from WT and PINK1-KO mice. GFAP protein expression was analyzed by Western blotting (left panel). Actin was used as a loading control. Phase contrast and GFAP antibody-stained fluorescence images were taken using a Zeiss microscope. (**C**, **G**) Proliferation was assayed in astrocytes prepared from WT and PINK1-KO mice (C, D) or PINK1-knockdown astrocytes (**E**–**G**). PINK1-knockdown astrocytes were prepared by transfecting WT astrocytes with PINK1-specific siRNA (siPINK1) as described in “Materials and Experimental Methods.” Nontargeting siRNA (siNT) was used as a control. The knockdown of PINK1 expression was confirmed by RT-PCR (**E**). After treating cells with serum-free medium for 24 h, proliferation was induced by 10 ng/mL EGF (C, F) or 10% FBS (D, G) for 24 h and then assayed by measuring [^3^H]-thymidine incorporation (C and D, left panels and F) or counting cells (C, D, and G, right panels) at the indicated times. Data are means ± SEMs of three sample (*P < 0.05; **P < 0.01). Data shown are representative of at least three independent experiments. Scale bar: 50 μm. [Color figure can be viewed in the online issue, which is available at http://wileyonlinelibrary.com.]

Astrocytes were cultured from the cerebral cortices of 1–3-day-old mice. Briefly, cerebral cortices were isolated and triturated with a glass pipette into single cells in Dulbecco's Modified Eagle Medium (DMEM) (Hyclone, Logan, UT) containing 10% FBS (Gemini Biological Products, Calabasas, CA). Dissociated cells were plated in 75-cm^2^ T-flasks and cultured until reaching confluence (2–3 weeks). After removing microglia by gently shaking, cells were harvested with 0.1% trypsin and re-plated for experiments. The purity of astrocytes, greater than 95%, and devoid of microglia was confirmed by immunocytochemistry using antibodies against glial fibrillary acid protein (GFAP, an astrocyte marker), and Iba-1 (a microglia marker; Supp. Info. Fig. 1).

### Immunocytochemistry

Astrocytes were fixed with 4% paraformaldehyde at room temperature for 20 min, washed with PBS, and incubated with 1% BSA and 0.1% Triton X-100 in PBS for 30 min. Next, cells were incubated overnight at 4°C with an anti-GFAP antibody, washed with PBS, and then incubated with fluorescein-conjugated secondary antibody (Invitrogen) for 2 h at room temperature. Cells were then washed, coverslip-mounted in Vectashield mounting medium containing the nuclear dye, 4′,6-diamidino-2-phenylindole (Vector laboratories, Burlingame, CA), and examined under an Axiovert 200M microscope (Carl Zeiss).

### RT-PCR and Q-PCR

Total RNA was isolated using RNAzol B (iNtRON, Sungnam, Korea), and cDNA was prepared using Avian Myeloblastosis Virus reverse transcriptase (Promega, Madison, WI) according to the manufacturers' instructions. PINK1 and glyceraldehyde 3-phosphate dehydrogenase (GAPDH) were amplified by RT-PCR using the following primer pairs: PINK1, 5′-GAGGGCGTGGACCATCTG-3′ (sense) and 5′-CTCGCCCCAGAGGCTTAAG-3′ (antisense); GAPDH, 5′-TGTTCCTACCCCCAATGTGT-3′ (sense) and 5′-TGTG-AGGGAGATGCTCAGTG-3′ (antisense). Amplified products were separated by electrophoresis on 1.5% agarose gels, and detected under ultraviolet light. EGFR and GADPH transcript levels were measured by quantitative PCR (Q-PCR) using a RotoGene thermocycler (Corbett Research, Sydney, Australia) and a KAPA SYBR FAST qPCR kit (Kapa Biosystems, Boston, MA), and the following primer pairs: EGFR, 5′-AGGCACAAGTAACAGGCTCAC-3′ (sense) and 5′-AAGGTCGTAATTCCTTTGCAC-3′ (antisense); GAPDH, 5′-GCCTTCCGTGTTCCTACC-3′ (sense) and 5′-CCTCAGTGTAGCCCAAGATG-3′ (antisense). The cycle threshold (Ct) for the EGFR transcript was normalized to the average Ct for transcripts of the housekeeping gene, GAPDH, amplified in each reaction. Relative quantitation of normalized transcript abundance was determined using the comparative Ct method (ΔΔCt), as described by the manufacturer.

### siRNA Transfection

The expression of target proteins (PINK1, p38 MAPK, and EGFR) was knocked down by transiently transfecting astrocytes with specific small interfering RNAs (siRNA; Genolution Pharmaceuticals, Seoul, Korea), as follows: PINK1 siRNA, 5′-GCGAAGCCAUCUUAAGCAAUU-3′; p38 siRNA #1, 5′-GACUGUGAGCUCAAGAUUCUU-3′; p38 siRNA #2, 5′-CGUUUCAGUCCAUCAUUCAUU-3′; p38 siRNA #3, 5′-GCAAGAAACUACAUUCAGUUU-3′; EGFR siRNA #1, 5′-CCAUCAAGGAGUUAAGAGAUU-3′; and EGFR siRNA #2, 5′-GAAUAUUAAGCAGCAUUUAUU-3′. For transfections, the medium was replaced with Opti-MEM (Invitrogen) and astrocytes were treated with 10 nM siRNA and RNAiMAX transfection reagent, according to the manufacturer's instructions (Invitrogen) for 5 days. Knockdown of targets was confirmed by RT-PCR (PINK1) and Western blotting (p38, EGFR), respectively.

### Proliferation Assay

Astrocyte proliferation was determined by assaying [^3^H]-thymidine (Perkin Elmer, Boston, MA) incorporation intoDNA. Astrocytes were plated in 24-well plates (8 × 10^4^ cell/well), incubated overnight, and starved in serum-free DMEM for 24 h. Cells were then treated with 10% FBS or 10 ng/mL EGF for 24 h in the presence of 1 μCi/mL [^3^H]-thymidine, washed twice with PBS, and lysedwith 0.1N NaOH. Radioactivity was determined using a β-counter (Packard Instruments, Downers Grove, IL).

### Measurement of Mitochondrial-Membrane Potential and Intracellular Reactive Oxygen Species (ROS)

Astrocytes were plated in 6-well plates (4 × 10^5^ cells/well) and incubated until reaching confluence. Mitochondrial membrane potential, mitochondrial mass, and intracellular ROS were monitored by loading cells for 30 min with 125 nM MitoTracker Red CMXRos, 125 nM MitoTracker Green FM, or 10 μM carboxyl-H2DFFDA, respectively, as described previously (Zhou et al., 2011). Cells were washed twice with PBS and detached with 0.1% trypsin; fluorescence intensities of detached cells were analyzed with a fluorescence-activated cell sorter (FACS; B-D FACS Systems, Sunnyvale, CA).

### Measurement of ATP Production

ATP generation was measured using an ATP Determination Kit (Invitrogen), as recommended by the manufacturer. Briefly, astrocytes were washed twice with ice-cold PBS and lysed with ATP Assay Buffer (Bio Vision, Mountain View, CA). After centrifugation at 13.4*g* for 5 min, the supernatant containing ATP was added to a bioluminescence reaction mix containing firefly luciferase and its substrate, D-luciferin. The amount of ATP in the supernatant was measured using a Victor 3 1420 multilabel counter (Perkin Elmer Life and Analytical Sciences, Shelton, CT).

### Glucose Uptake Assay

Glucose uptake was assayed using [^3^H]-2-deoxyglucose (Perkin Elmer). Astrocytes were plated in 24-well plates (8 × 10^4^ cells/well) and cultured overnight. Cells were then starved by incubating with glucose-free DMEM (Invitrogen) for 3 h and incubated with 1 μCi/mL of [^3^H]-2-deoxyglucose for 20 min. Subsequently, cells were washed twice with PBS and lysed with 0.1N NaOH. Radioactivity was determined as counts per minute with a β-counter (Packard Instruments).

### Western Blot Analysis

Astrocytes were washed twice with cold PBS and lysed on ice with modified RIPA buffer (50 mM Tris-HCl, pH 7.4, 1% NP-40, 0.25% Na-deoxycholate, 150 mM NaCl, 1 mM Na_3_VO_4_, and 1 mM NaF) containing protease inhibitors (2 mM phenylmethylsulfonyl fluoride [PMSF], 10 μg/mL leupeptin, 10 μg/mL pepstatin, and 2 mM EDTA) and phosphatase inhibitor cocktail (GenDEPOT, Barker, TX). Proteins in whole-cell extracts were resolved by sodium dodecyl sulfate-polyacrylamide gel electrophoresis and transferred to nitrocellulose transfer membranes. Membranes were incubated with primary antibodies specific for phosphor-AKT (p-AKT), phosphor-p38 (p-p38), phosphor-signal transducer, and activator of transcription-3 (p-STAT3), phosphor-ERK1/2 (p-ERK1/2), EGFR, AKT, p38, GFAP, and actin. Antibodies specific for GFAP and actin were from Sigma (St. Louis, MO), and Santa Cruz Biotechnology (Santa Cruz, CA), respectively; and antibody specific for PINK1 was from Abcam (Cambridge, MA); all other antibodies were from Cell Signaling Technology (Beverly, MA). Membranes were incubated with peroxidase-conjugated secondary antibodies (Jackson ImmunoResearch, West Grove, PA), and immunoreactive proteins were visualized usingan enhanced chemiluminescence system (Daeil Lab, Seoul, Korea).

### Scratch-Wound Healing Assay

Astrocytes were plated and incubated until they reached confluence. Cell monolayers were scratched with a blue (1-mL) pipet tip, washed with culture media, and then incubated for 1–5 days to allow recovery. Scratched areas before and after recovery were measured using Axiovision 4.1 software (Carl Zeiss, Göttingen, Germany).

### Statistical Analysis

All data presented in this study are representative of at least three independent experiments. The statistical significance of differences between mean values was assessed by Student's t-test. For comparisons of more than two groups, we used one-way ANOVA with Duncan's *post-hoc* test.

## RESULTS

### PINK1 Deficiency Down-Regulates Astrocyte Proliferation

To assess the potential role of PINK1 in astrocytes, we analyzed differences in the properties of astrocytes cultured from WT and PINK1-KO mice (Xiong et al., 2009). PINK1 deficiency in cultured astrocytes was confirmed by examining the levels of PINK1 mRNA and protein by RT-PCR and Western blot, respectively (Fig. 1A). Astrocytes prepared from WT and PINK1-KO mice showed no differences in morphology or GFAP expression levels (Fig. 1B). However, we foundthat the proliferative capacity of PINK1-KO astrocytes was significantly decreased compared with that of WT astrocytes (Fig. 1C,D). In these experiments, astrocytes were incubated with serum-free media for 24 h and then proliferation was measured by [^3^H]-thymidine incorporation and cell counting after exposure to EGF (10 ng/mL), a well-known astrocyte mitogen, or FBS (10%). Similar results were obtained in WT astrocytes transfected with PINK1 siRNA (Fig. 1E–G). RT-PCR confirmed that PINK1 siRNA, but not nontargeting (NT) siRNA, reduced PINK1 expression (Fig. 1E). In response to EGF and FBS treatment, proliferation, determined by counting cell number and/or [^3^H]-thymidine incorporation, was decreased in PINK1 siRNA-treated astrocytes (Fig. 1F,G). Collectively, these results suggest that PINK1 positively regulates astrocyte proliferation.

### Altered AKT and p38 Activation, and EGFR Expression in PINK1-KO Astrocytes

**FIGURE 2:**
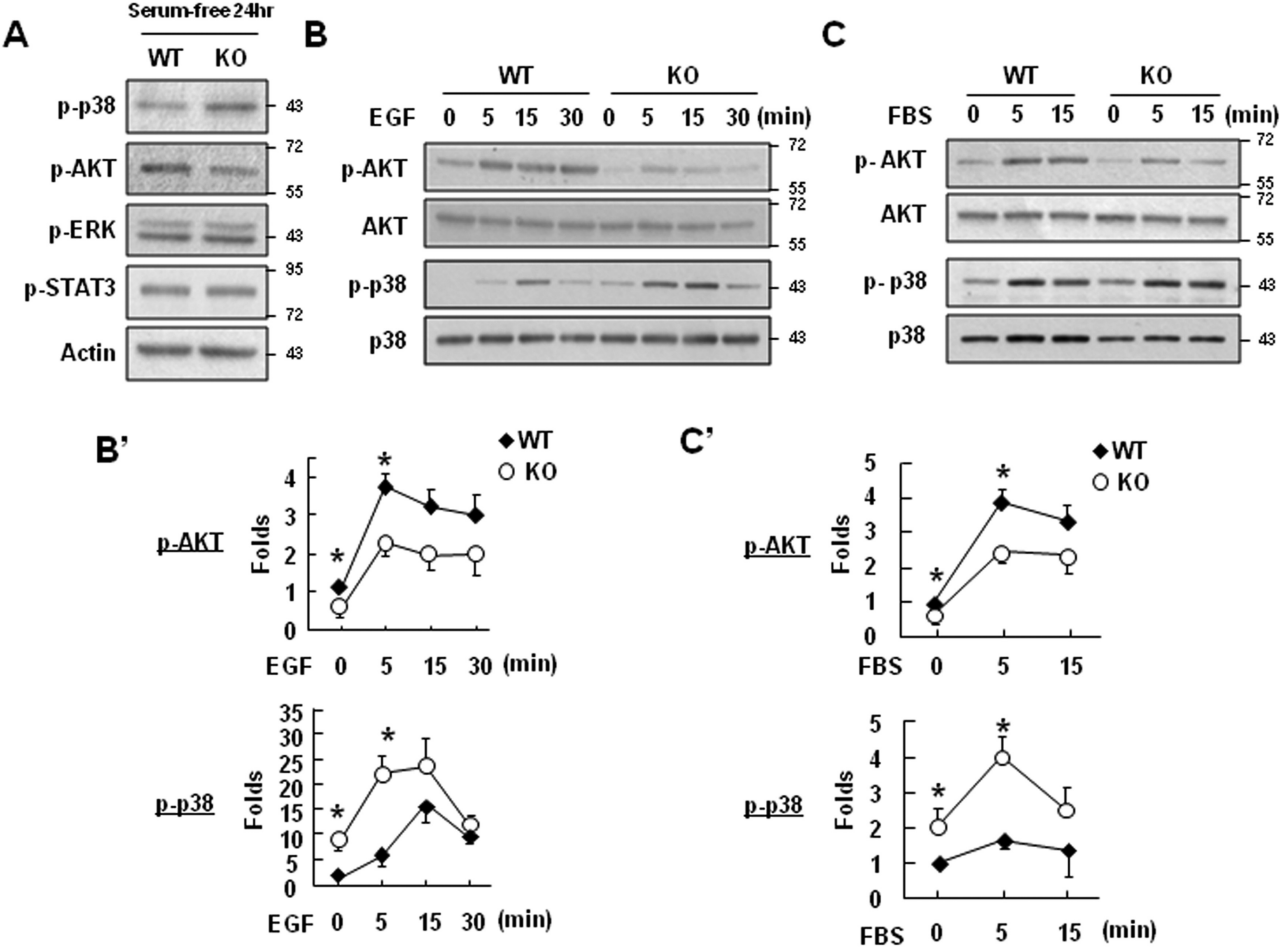
PINK1 deficiency attenuates AKT activation but enhances p38 MAPK activation. (**A**) Activation levels of AKT, p38, ERK, and STAT3 were assayed in WT and PINK1-KO astrocytes after cells were incubated for 24 h in serum-free DMEM. (**B**, **C**) Cells were treated with EGF (10 ng/mL) or FBS (10%) for the indicted times after incubation in serum-free DMEM for 24 h. Levels of p-AKT, p-p38, p-ERK, and p-STAT3 were determined by Western blotting using the corresponding phosphospecific antibodies. Actin (A), and AKT and p38 (B, C) were used as loading controls. (**B′**, **C′**) Band intensities of p-AKT and p-p38 in (B, C) were quantified. Values are means ± SEMs of three samples (*P < 0.05). Data shown are representative of at least three independent experiments.

Next, we examined signaling molecules that might mediate the differences in astrocyte proliferation in WT and PINK1-KO cells. Several signaling molecules, including ERK1/2, AKT, p38, and STAT3 are known to mediate astrocyte proliferation in response to growth factors and/or injury (Bajetto et al., 2001; Fraser et al., 2004; Herrmann et al., 2008; Tsuda etal., 2011; Xu et al., 2007). In astrocytes cultured in serum-free media for 24 h, the levels of p-AKT were lower in PINK1-KO astrocytes than in WT astrocytes, whereas levels of p-p38 were higher in PINK1-KO astrocytes; levels of p-STAT3 and p-ERK1/2 did not differ according to PINK1 status (Fig. 2A). PINK1 siRNA also decreased levels of p-AKT and EGFR, and increased levels of p-p38 in WT astrocytes (Supp. Info. Fig. 2). In response to EGF, p-AKT and p-p38 levels increased in both WT and PINK1-KO astrocytes, but the increase in p-AKT was smaller in PINK1-KO astrocytes than in WT astrocytes, whereas the increase in p-p38 was larger in PINK1-KO astrocytes (Fig. 2B,B′). Levels of p-STAT3 and p-ERK1/2 following EGF treatment were not different between PINK1-KO and WT astrocytes (data not shown). In response to FBS, levels of p-p38 were higher but levels of p-AKT were lower in KO astrocytes (Fig. 2C,C′).

**FIGURE 3:**
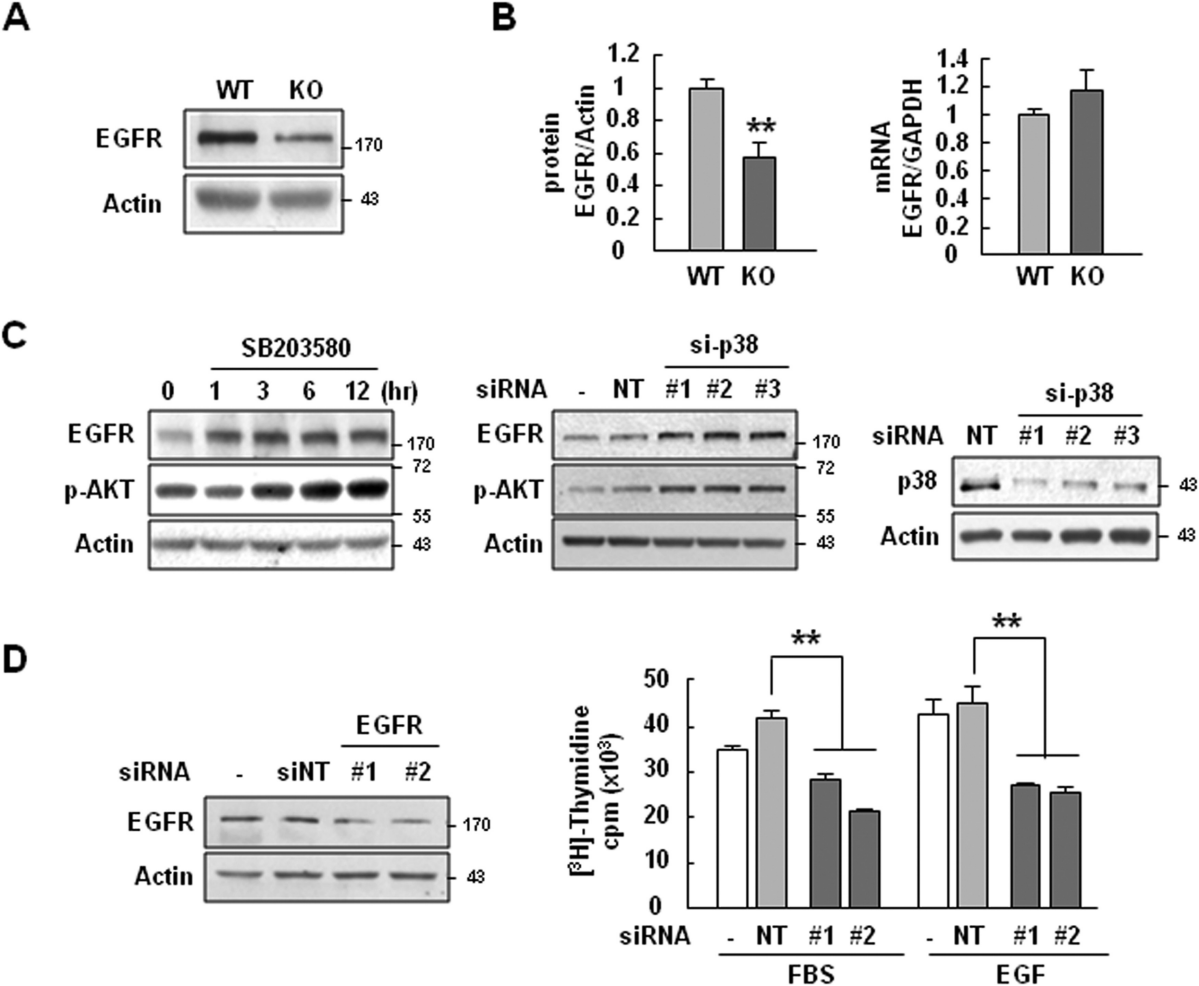
p38 MAPK regulates EGFR expression and AKT activation. (**A**, **B**) EGFR protein (A) and mRNA (B, right panel) expression levels were compared in WT and PINK1-KO astrocytes using Western blotting and Q-PCR, respectively. Actin was used as a loading control. EGFR band densities were quantified and normalized to those of actin (B, left panel). (**C**) PINK1-KO astrocytes were treated with SB203580 (10 μM) for the indicated times (left panel) or transfected with nontargeting (NT) or p38-specific siRNA (10 nM) for 5 d (middle and right panels). Expression levels of EGFR and p38, and p-AKT levels were determined by Western blotting. Actin was used as a loading control. (**D**) EGFR-knockdown astrocytes were prepared by transfecting WT astrocytes with EGFR-specific siRNA as described in “Materials and Experimental Methods.” Nontargeting siRNA (siNT) was used as a control. EGFR-knockdown was confirmed with Western blooting (left panel). Proliferation was assayed in EGFR-knockdown WT astrocytes (right panel). Proliferation induced by 10 ng/mL EGF or 10% FBS was assayed by measuring [^3^H]-thymidine incorporation. Data in (B) and (D) are means ± SEMs of three samples (*P < 0.05; **P < 0.01). Data shown are representative of at least three independent experiments.

Since activation of p38 causes degradation of EGFR in epithelial cells and HeLa cells (Frey et al., 2006; Vergarajauregui et al., 2006), we examined possible down regulation of EGFR in PINK1-KO astrocytes. As predicted, EGFR protein levels (Fig. 3A), but not mRNA levels (Fig. 3B, right panel), were significantly reduced in PINK1-KO astrocytes compared with WT astrocytes. Using p38 siRNA and a chemical inhibitor of p38 (SB203580), we found that attenuation of p38 expression and inhibition of p38 activity restored EGFR expression in PINK1-KO astrocytes (Fig. 3C). In addition, both p38 siRNA and SB203580 enhanced basal AKT activation (Fig. 3C). We further examined whether the reduced EGFR expression could underlie the defective proliferative response to both FBS and EGF. WT astrocytes treated with two different EGFR-specific siRNAs showed decreased proliferation following stimulation with either FBS or EGF (Fig. 3D), suggesting that decreased EGFR expression was responsible for the defective proliferative response to both stimuli. These results suggest that PINK1 deficiency increases p38 activation, which, in turn, inhibits AKT activation and EGFR expression, leading to the proliferation defects in PINK1-KO astrocytes.

### Defective Mitochondrial Function and Glucose Uptake Are Linked to the Proliferation Defect in PINK1-KO Astrocytes

**FIGURE 4:**
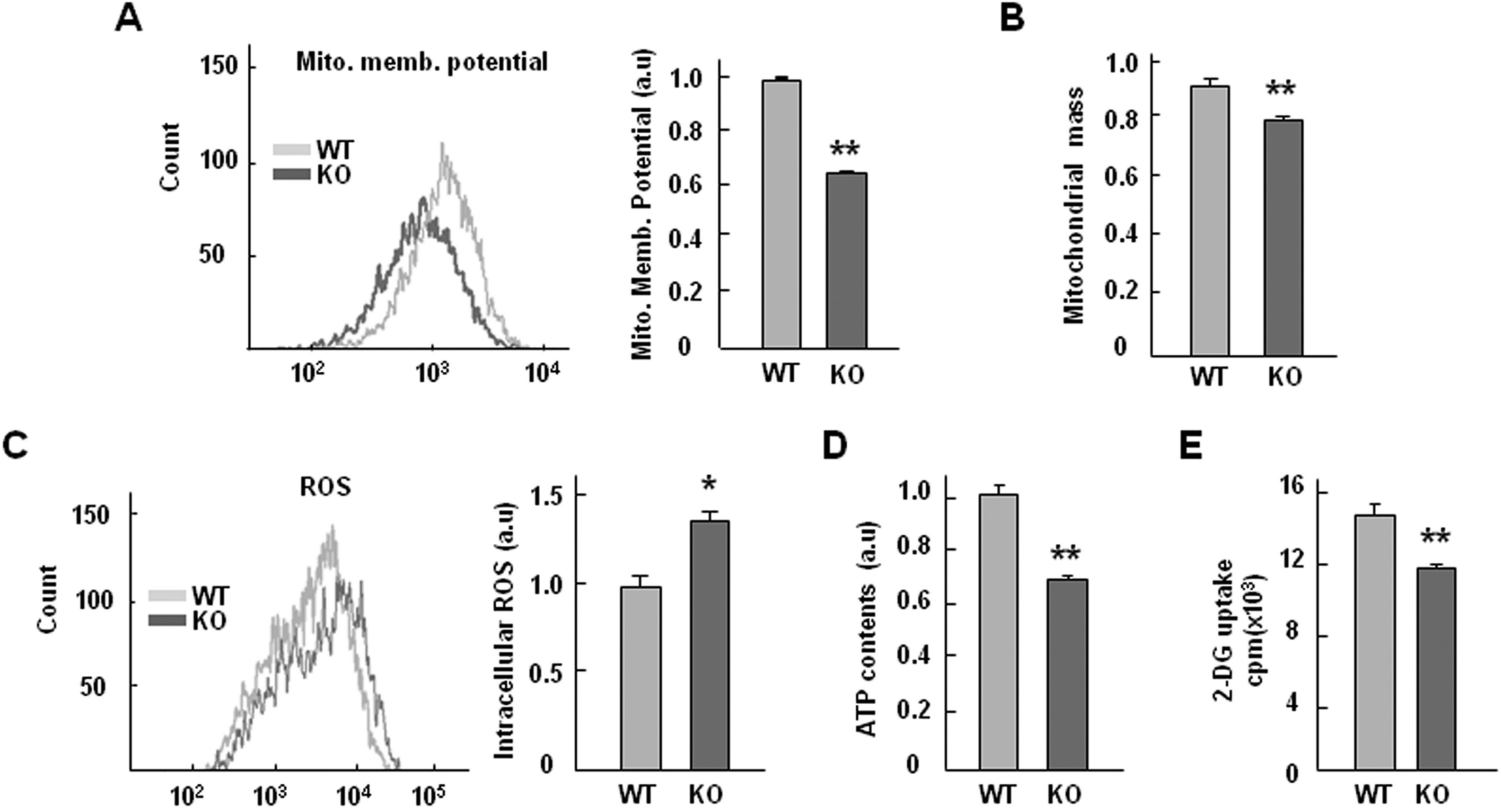
Mitochondrial dysfunction in PINK1-KO astrocytes. (**A–C**) WT and PINK1-KO astrocytes were incubated with 125 nM MitoTracker Red CMXRos (A), 125 nM MitoTracker Green FM (B), and 10 μM carboxyl-H2DFFDA (C) for 30 min to assay mitochondrial membrane potential, mitochondrial mass, and intracellular ROS, respectively. Fluorescence intensities were determined by FACS analysis. (**D**) ATP production, measured as described in “Materials and Experimental Methods.” (**E**) Glucose uptake, assayed using [^3^H]-2-deoxyglucose as described in “Materials and Experimental Methods.” Data are means ± SEMs of three samples (*P < 0.05; **P < 0.01). Data shown are representative of at least three independent experiments.

It has been reported that PINK1 deficiency causes mitochondrial defects in neurons (Gandhi et al., 2009). Therefore, we examined whether PINK1-KO astrocytes also have defects in mitochondrial function, and whether these defects could be involved in the decreased ability to proliferate. First, mitochondrial membrane potential and mitochondrial mass were measured using MitoTracker Red CMXRos, MitoTracker Green FM, and FACS analysis. As predicted, mitochondrial membrane potential and mitochondrial mass were significantly reduced in PINK1-KO astrocytes com- pared with WT astrocytes (Fig. 4A,B). In addition, intracellular ROS levels, analyzed with carboxyl-H_2_DFFDA and FACS,were significantly increased in PINK1-KO astrocytes (Fig. 4C). Because mitochondria are the major source of ATP production in cells, we measured ATP content to determine whether mitochondrial dysfunction in PINK1-KO astrocytes affected ATP production. As predicted, ATP content was also decreased in PINK1-KO astrocytes (Fig. 4D). Because ROS generation due to mitochondrial dysfunction eventually reduce glucose uptake (Gandhi et al., 2009), we further analyzed glucose uptake using [^3^H]-2-deoxyglucose. As shown in Fig. 4E, the mitochondrial dysfunction associated with PINK1 deficiency did indeed cause a reduction in glucose uptake in astrocytes.

**FIGURE 5:**
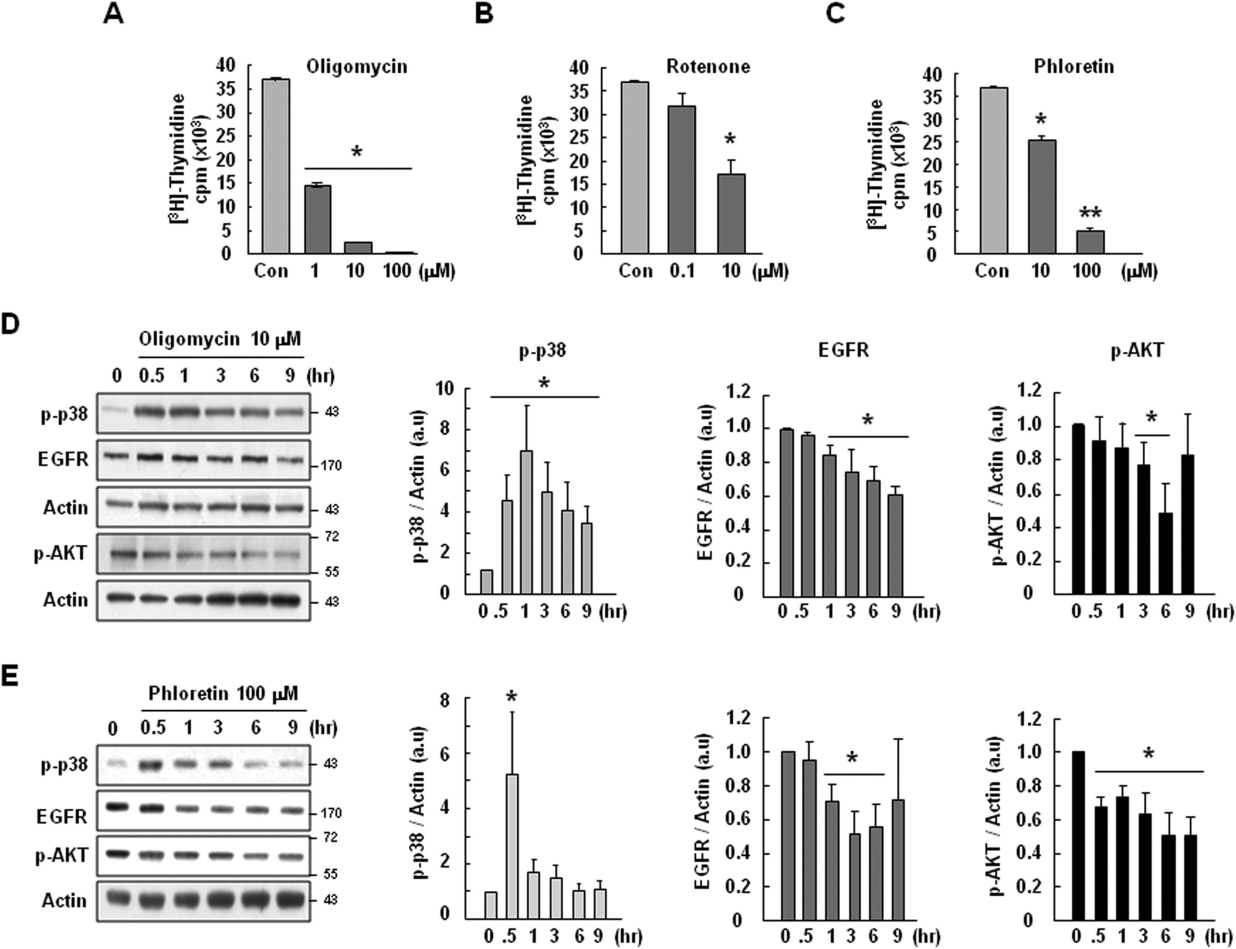
Mitochondrial dysfunction is associated with the defect in proliferation and altered EGFR expression, and AKT and p38 activation. (**A**–**C**) Proliferation of WT astrocytes was assayed in the presence of the indicated amount of oligomycin (A), rotenone (B), or phloretin (C). Proliferation was induced by incubating with 10% FBS for 48 h, and measured by assaying [^3^H]-thymidine (1 μCi/mL) incorporation. (**D**, **E**) WT astrocytes were treated with oligomycin (10 μM, D) or phloretin (100 μM, E) for the indicated times after 24 h incubation in serum-free medium. EGFR expression and AKT and p38 activation were detected by Western blotting. Band densities of EGFR, p-AKT and p-p38 were quantified (D, E, right panel). Data are means ± SEMs of three sample (*P < 0.05; **P < 0.01). Data shown are representative of at least three independent experiments.

We next examined whether defects in mitochondrial function were involved in the reduced proliferative capacity of PINK1-KO astrocytes. The direct mitochondrial toxins, rotenone (a complex 1 inhibitor), and oligomycin (an ATP synthase inhibitor) induced a concentration-dependent reduction in WT astrocyte proliferation (Fig. 5A,B). Phloretin, an inhibitor of glucose transporter 1 (GLUT1), also de-creased the proliferation of WT astrocytes in a concentration-dependent manner (Fig. 5C). The absence of toxicity of these reagents was confirmed by LDH assay (Supp. Info. Fig. 3).

Next, we analyzed whether mitochondrial dysfunction affects proliferation-regulating signaling molecules by examining the effects of oligomycin and phloretin on p38 activation, AKT phosphorylation, and EGFR expression. Oligomycin enhanced p38 activation but decreased AKT activation and EGFR expression within 0.5–3 h after treatment, and the effect was sustained for up to 6–9 h (Fig. 5D). Phloretin treatment produced similar results: p38 activation was enhanced, and AKT activation and EGFR expression were decreased (Fig. 5E). Taken together, these results suggest that mitochondrial dysfunction in PINK1-KO astrocytes contributes to the proliferation defect through regulation of p38 and AKT activation, and EGFR expression.

### Delayed Wound Healing in PINK1-KO Astrocytes

**FIGURE 6:**
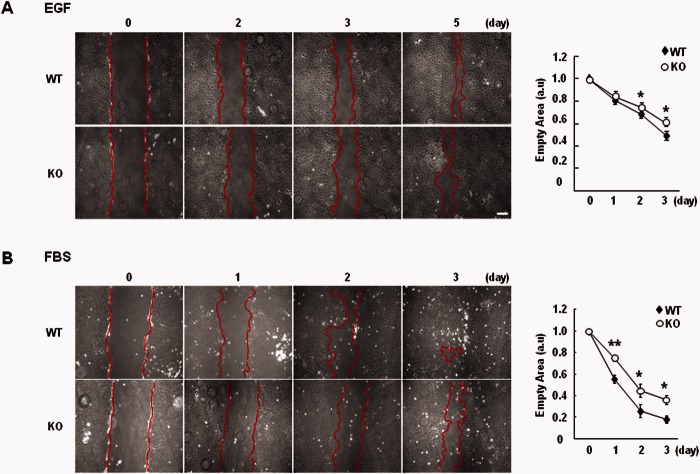
Wound-healing processes are delayed in PINK1-KO astrocytes. WT and PINK1-KO astrocytes were plated on 6-well plates, cultured until reaching confluence, wounded as described in “Materials and Experimental Methods,” and cultured with DMEM containing 10 ng/mL EGF (**A**) or 10% FBS (**B**). Astrocyte-absent areas were measured on day 0 (immediately after scratch damage) and days 1, 2, and 3 (right panels of A, and B) using Axiovision 4.1 software. Data are means ± SEMs of three samples (*P < 0.05; **P < 0.01). Data shown are representative of at least three independent experiments. Scale bar: 200 μm. [Color figure can be viewed in the online issue, which is available at http://wileyonlinelibrary.com.]

Because astrocytes proliferate and participate in repair processes in the injured brain (Sofroniew, 2009), we next compared the wound healing capacity of PINK1-KO and WT astrocytes. In a scratch–wound healing assay, a model used to mimic central nervous system (CNS) injury, astrocytes filled the astrocyte-absent areas after the wound was made (Fig. 6). As hypothesized based on the proliferation defect in PINK1-KO astrocytes, wound healing was delayed in PINK1-KO cells compared with WT cells in the presence ofeither EGF or FBS (Fig. 6A,B). Next, we examined whether delayed wound healing in PINK1-KO astrocytes was due to delayed migration ability. In migration assays using transwells, the number of astrocytes that migrated to the bottom of the transwell was not different between PINK1-KO and WT astrocytes (Supp. Info. Fig. 4A). In addition, in the presence of cytosine arabinoside (AraC), an inhibitor of proliferation, PINK1-KO and WT astrocytes showed similar wound-healing ability, which suggests that migration ability, may not be different in PINK1-KO and WT astrocytes (Supp. Info. Fig. 4B). Instead, PINK1-KO astrocytes may not efficiently proliferate to fill the injury sites.

## DISCUSSION

Pathophysiological mechanisms of PD have long been the focus of intensive investigations, and several familial PD-related genes, including PINK1, have been identified. Although cultured neurons from PINK1 mutant animals and/or cell lines in which PINK1 expression is manipu- latedbecome vulnerable to several insults, animal models thatcarry a PINK1 mutation do not develop PD-like symptoms, such as degeneration of dopaminergic neurons, motor deficits, and Lewy body formation (Deng et al., 2005; Haque et al., 2008). Consistent with these observations, the PINK1-KO mice used in this study and other studies showed no symptoms of PD (Chen et al., unpublished; Gispert et al., 2009; Kitada etal., 2007). The differential effects of manipulating PD-related genes *in vitro* and *in vivo* are not confined to PINK1. Other PD-related genes, including DJ-1, parkin, α-synuclein, and LRRK2, also show different effects on neuronal viability *in vitro* and *in vivo*. In the case of DJ-1, parkin and PINK1, even triple KO did not induce neurotoxicity *in vivo* (Kitada et al., 2009). These findings strongly suggest that environmental factors cooperate with genetic factors in the development and progression of PD although we could not exclude the difference between rodents and humen, in respect of metabolism, life span, and development of pathological condition, etc (Demetrius, 2005; Fougerousse et al., 2000; Ginis et al., 2004; Ishikawa et al., 2010; Mestas and Hughes, 2004).

Astrocytes, which constitute a majority of cells in the brain, could be the most important regulator of the brain microenvironment. Accordingly, impaired astrocyte function is closely related to the development of amyotrophic lateral sclerosis and epilepsy in animal models (Barbeito etal., 2004; Coulter and Eid, 2012; Hinterkeuser et al., 2000). In the intact brain, astrocytes provide neurons with nutrients and growth factors, and maintain homeostasis of extracellular glutamate and K^+^ levels (White and Jakeman, 2008). In injury states, astrocyte function becomes more important since homeostasis of the brain microenvironment is disrupted. In the injured brain, astrocytes function to suppress oxidative stress (Fernandez-Gonzalez et al., 2000) and inflammatory responses (Kim et al., 2010; Min et al., 2006), as well as to remove increased extracellular glutamate and K^+^. Astrocyte proliferation is also important in the injured brain (Bush et al., 1999; Faulkner et al., 2004; Myer et al., 2006; Voskuhl et al., 2009; White and Jakeman, 2008). Astrocytes proliferate and isolate injury sites, serving to prevent propagation of harmful factors to the surrounding area (Costa et al., 2002; Tom et al., 2004). Astrocytes also regulate revascularization and remyelination in the injured brain (Talbott et al., 2005; Whetstone et al., 2003). Accordingly, intrathecal infusion of FGF-2 or EGF increases glial proliferation and improves functional recovery in spinal cord injury (Kojima and Tator, 2002). In addition, in a spinal cord injury model using the mouse strain, 129X1/SvJ, whose astrocytes readily invade lesion sites and proliferate, axons grow rapidly into the lesion core (Ma et al., 2001; White and Jakeman, 2008). We also found that, in the injured brain, astrocytes proliferate and fill the damaged area, and neurite outgrowth and blood vessel regeneration occur (unpublished observation). Important roles of proliferating astrocytes have also been inferred from experiments using GFAP-thymidine kinase (TK) mice, in which proliferating astrocytes are selectively depleted. In models of spinal cord injury, trauma and stab wound, leukocyte infiltration, disruption of the blood brain barrier, and CNS tissue damage are significantly increased in GFAP-TK mice (Bush et al., 1999; Faulkner et al., 2004; Kojima and Tator, 2002; Ma et al., 2001; Myer et al., 2006; Voskuhl et al., 2009; White and Jakeman, 2008). Astrocyte proliferation has been detected in the brains of PD patients and in MPTP- and 6-hydroxydapamine-induced PD animal models (Kohutnicka et al., 1998; Stromberg et al., 1986). Furthermore, it has been reported that, in PD and Parkinsonian syndromes, astrocytes do not properly exert their beneficial roles due to a defect in their proliferative response to early damage (Mirza etal., 2000; Song et al., 2009). In this study, we found that PINK1 KO causes defects in astrocyte proliferation (Fig. 1) and delays wound-healing processes (Fig. 6). PINK1 siRNA also attenuated astrocyte proliferation and wound healing processes (Supp. Info. Fig. 2). Therefore, PINK1-KO astrocytes may delay isolation of injury sites and have insufficient capacity to maintain microenvironmental homeostasis and repair injury sites.

The next question we addressed was how PINK1 deficiency reduces astrocyte proliferation. It has been reported that PINK1 mutation, knock-down, or knock-out decreased mitochondrial respiration, mitochondria membrane potential, and ATP generation, and increased ROS, which result in an increased susceptibility to apoptosis in various types of cells including neurons, skeletal muscles, and fibroblasts (Abramov et al., 2011; Beilina et al., 2005; Gandhi et al., 2009; Liu etal., 2009; Sim et al., 2006; Yao et al., 2011). In astrocytes, PINK1-KO causes mitochondrial dysfunction, as demonstrated by reduced mitochondrial mass, increased mitochondrial membrane potential and intracellular ROS, and decreased ATP production and glucose uptake (Fig. 4). PINK1 siRNA similarly reduced mitochondrial function (Supp. Info. Fig. 2). The mitochondrial defect appeared to cause the proliferation defect, since mitochondrial toxins (rotenone and oligomycin) and a glucose-uptake inhibitor (phloretin) also reduced astrocyte proliferation in WT astrocytes (Fig. 5A–C).

Mitochondrial dysfunction could affect signaling pathways that mediate astrocyte proliferation. In our study, PINK1 deficiency showed reduced AKT activation and EGFR expression, but increased p38 activation, compared with WT astrocytes (Figs. 2 and 3, and Supp. Info. Fig. 2). Mitochondrial toxins (oligomycin) and a glucose-uptake inhibitor (phloretin) also reduced AKT activation and EGFR expression, but increased p38 activation (Fig. 5D,E). Interestingly, there was crosstalk between AKT, EGFR, and p38: p38 reduced AKT activation and EGFR expression, as evidenced by the fact that a chemical inhibitor of p38 (SB203580) and p38-specific siRNAs increased AKT activation and EGFR expression (Fig. 3C). Consistent with this, it has been reported that p38 negatively regulates EGFR levels through endocytosis in epithelial cells and HeLa cells (Frey et al., 2006; Vergarajauregui et al., 2006). It has been reported that STAT3 KO astrocytes showed defect in proliferation and mitochondrial function (Sarafian et al., 2010). Furthermore, there is a crosstalk between JAK-STAT and PI3K/MAPK pathways (Gross et al., 2006; Hu et al., 2007; Platanias, 2003; Rawlings et al., 2004). However, STAT3 activation levels were not different in PINK1 WT and KO astrocytes (Fig. 2A). In astrocytes treated with EGFR-specific siRNA, FBS-induced astrocyte proliferation was reduced, indicating that FBS-induced proliferation of astrocytes was mediated, at least in part, by the EGFR (Fig. 3D). However, we still cannot exclude the possibility that expression of other growth factor receptors or downstream factors could be altered in PINK1-KO astrocytes. Altered activation of p38 and AKT has been reported in PD patients and PD animal models. Specifically, p38 activation is enhanced in PD patients and the brains of MPTP-treated mice (Boger et al., 2008; Ferrer et al., 2001; Karunakaran et al., 2008), and AKT activation is reduced in PINK1-null mutant drosophila (Tain et al., 2009). Therefore, p38 and AKT, and their downstream targets, could be linked to PD onset and progression.

In addition to PINK1, DJ-1 and Parkin also could regulate astrocyte function. It has been reported that DJ-1 knock-down in astrocytes decreased mitochondrial motility, enhanced rotenone-induced mitochondrial membrane potential, and reduced neuroprotection against rotenone (Larsen et al., 2011; Mullett and Hinkle, 2009). Mixed glia cells from Parkin KO mice showed reduced proliferation, increased proapoptotic protein expression, and increased susceptibility to neurotoxins (Solano et al., 2008). These evidences suggest that altered astrocyte functions due to mutation of PD-related genes contribute to cause or amplify pathogenesis of PD.

Here, we provide the first demonstration that PINK1 deficiency causes defects in astrocyte proliferation. We demonstrated corresponding changes in critical signal pathways, showing that PINK1 deficiency in astrocytes causes mitochondrial defects, which in turn leads the proliferation defect through changes in proliferation-regulating signaling pathways (p38 and AKT activation; EGFR expression). Therefore, our study implies that PINK1 could play a role in repair process in pathological situations, and suggest that PINK1 deficiency could result in abnormal tissue repair of the injured brain and increase the risk of PD.
