# Supplementary figures and images for: PINK1 Deficiency Attenuates Astrocyte Proliferation Through Mitochondrial Dysfunction, Reduced AKT and Increased p38 MAPK Activation, and Downregulation of EGFR
Source: Glia. 2013 Feb 26;61(5):800–12. doi: 10.1002/glia.22475 (PMC3657120; doi:10.1002/glia.22475)

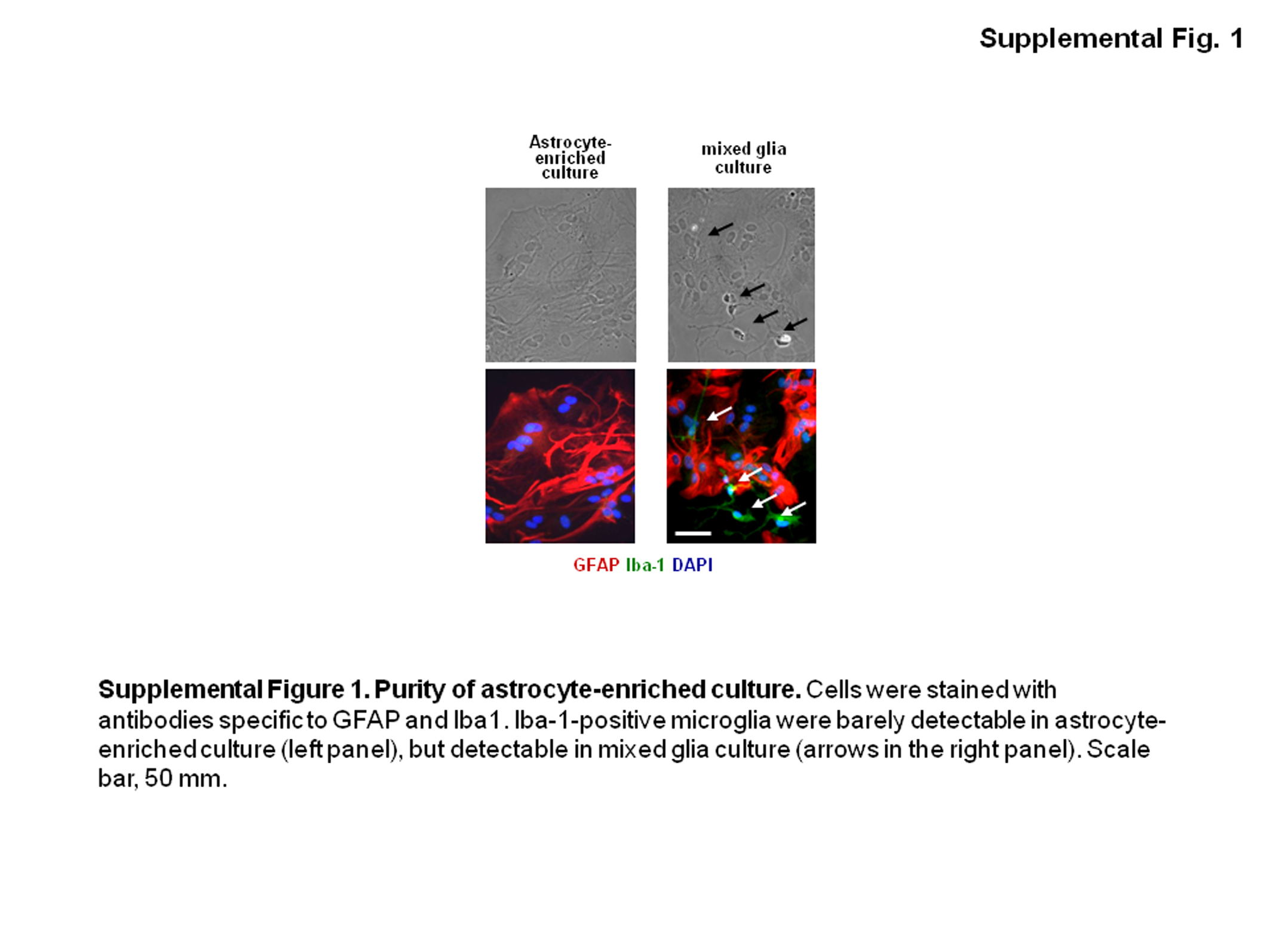

Supplement: Supplementary file 1 [file glia0061-0800-SD1.tif]

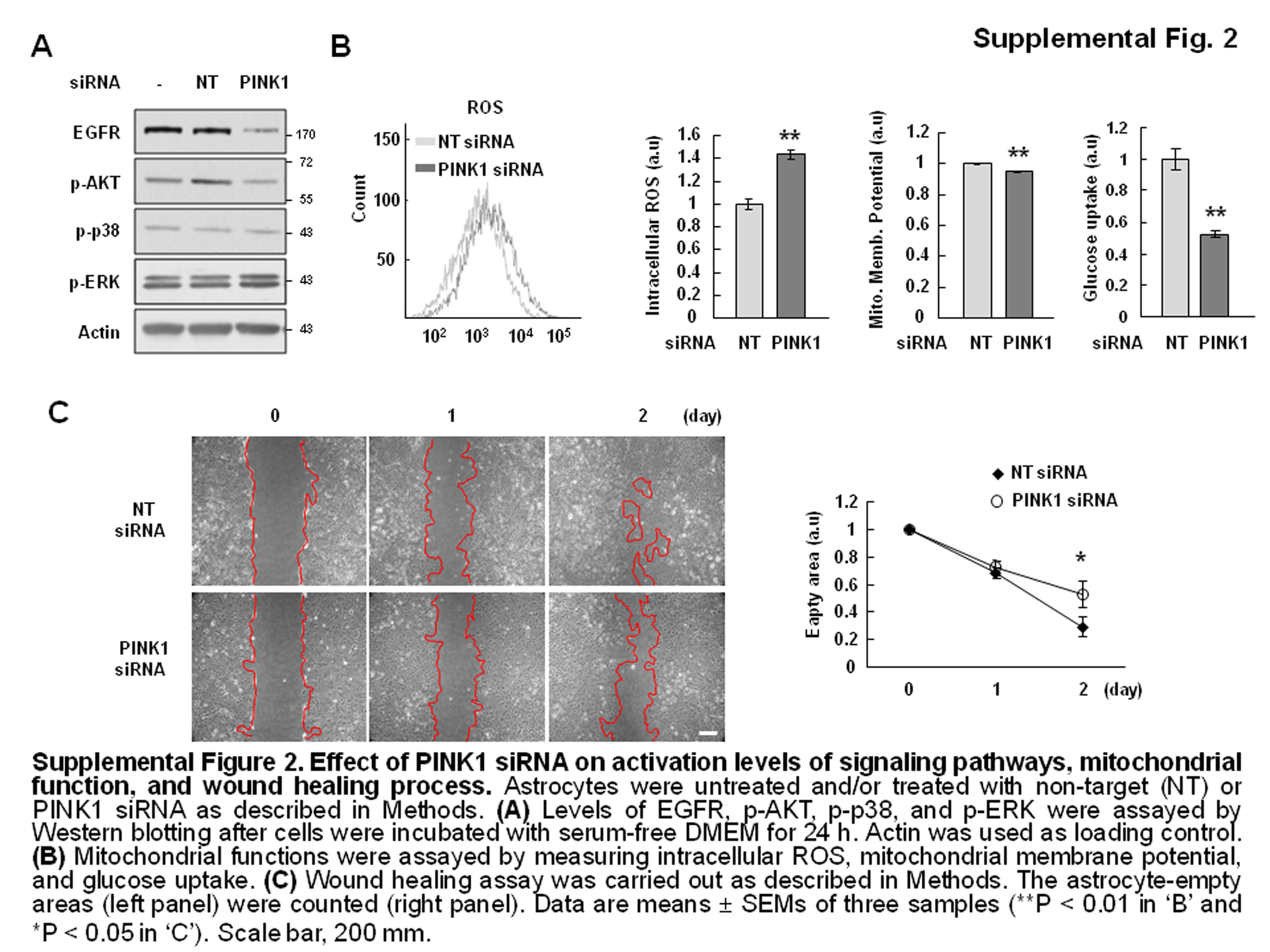

Supplement: Supplementary file 2 [file glia0061-0800-SD2.tif]

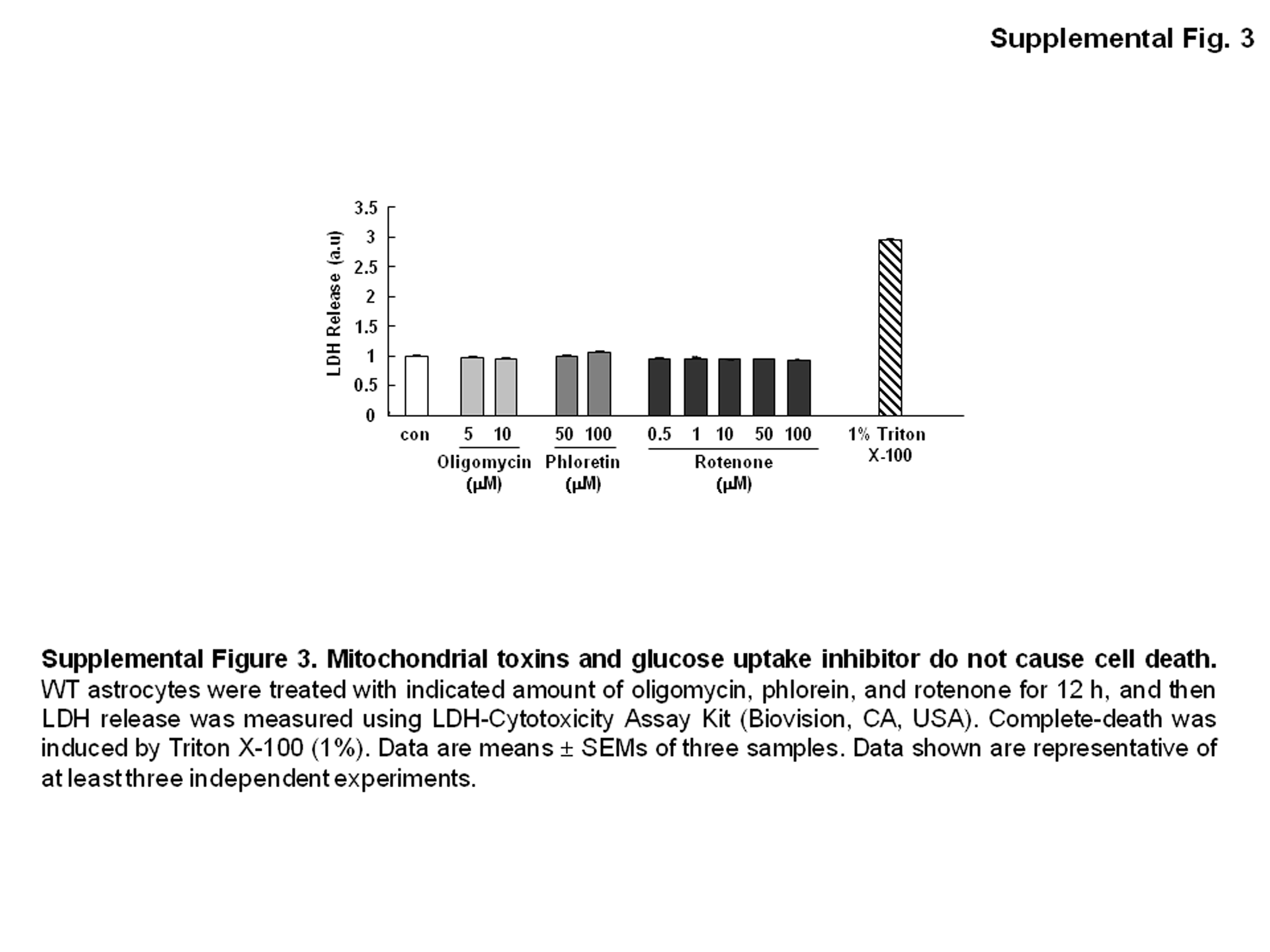

Supplement: Supplementary file 3 [file glia0061-0800-SD3.tif]

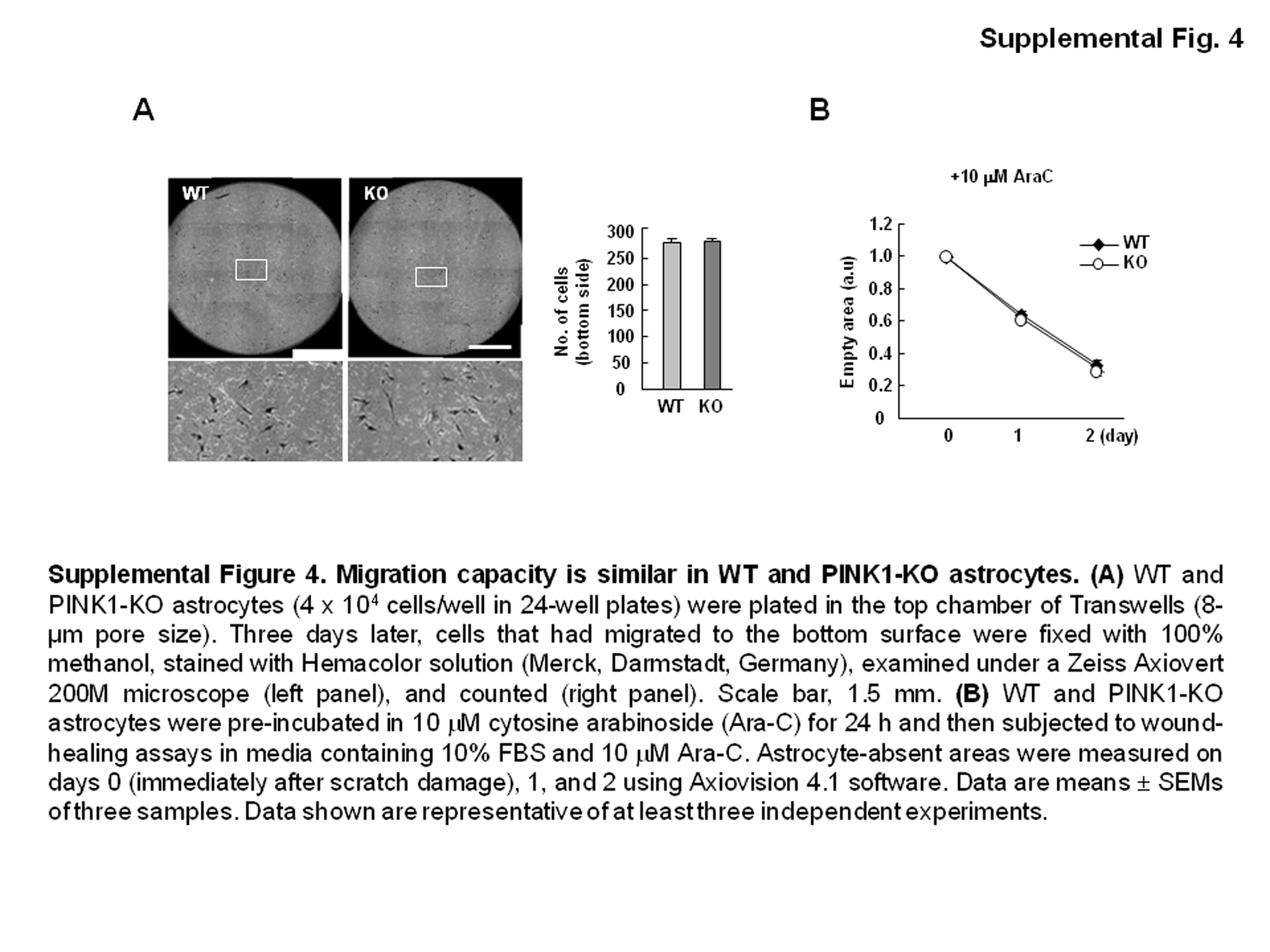

Supplement: Supplementary file 4 [file glia0061-0800-SD4.tif]
